# An acidophilic GH12 xyloglucanase from *Trichoderma asperellum* produces prebiotic oligosaccharides that promote probiotic growth

**DOI:** 10.3389/fnut.2026.1850050

**Published:** 2026-06-29

**Authors:** Fengzhen Zheng, Ruihao Zhang, Sicheng Rong, Huan Zhuang, Jiaqiang Wang, Abdul Basit

**Affiliations:** 1 Zhejiang International Joint Laboratory on Low-Carbon Pollution Control and Resource Utilization, College of Biological and Environmental Engineering, Zhejiang Shuren University, Hangzhou, China; 2 Department of ENT and Head and Neck Surgery, The Children's Hospital Zhejiang University School of Medicine, Zhejiang, Hangzhou, China; 3 Department of Biomedical Engineering, Whiting School of Engineering, Johns Hopkins University, Baltimore, MD, United States; 4 Department of Microbiology, University of Jhang, Jhang, Pakistan

**Keywords:** acidophilic tolerance, catalytic sites, prebiotics, structural analysis, *Trichoderma asperellum*, Xyloglucanase

## Abstract

Enzymatic transformation of lignocellulosic biomass offers an eco-friendly pathway to produce valuable *β*-oligoglucosides. In this study, an acidophilic GH12 xyloglucanase (TaXEG12) from *Trichoderma asperellum* ND-1 was overexpressed in *Komagataella phaffii*. TaXEG12 displayed maximum activity at 40 °C and a pH of 4.0, while retaining >80% activity in the presence of 1.71 M NaCl and 10% ethanol. Glu^140^ and Glu^227^ are important catalytic residues, while Asp^126^ plays an auxiliary role in xyloglucan degradation. The enzyme showed strict substrate specificity toward tamarind xyloglucan. Through fed-batch fermentation, the enzyme production of TaXEG12 improved by 28.5-fold (2914.5 ± 65.7 U/mL). Interestingly, TLC and ESI-MS analyses revealed that TaXEG12 could hydrolyze xyloglucan into xyloglucan oligosaccharides (XXXG, XXLG/XLXG, and XLLG). These xyloglucan oligosaccharides can promote the growth of *Lactobacillus bulgaricus*. Additionally, the synergy degrees of corn bran and apple pulp in the degradation by TaXEG12 and TrCel were 1.34 and 1.32, respectively. TaXEG12 displayed unique properties and could be a desirable catalyst for the more sustainable production of high-value oligosaccharides, which are significantly beneficial for human intestinal health.

## Graphical abstract

**Figure:**
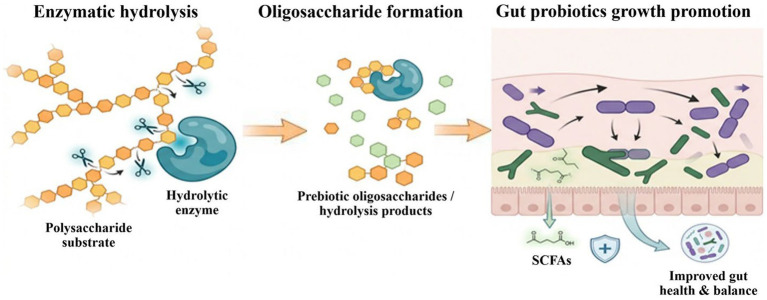

## 1 Introduction

Hemicellulosic biomass is the second most abundant renewable polysaccharide in plants, and its utilization contributes to sustainable biotechnology fields (1). Xyloglucan is the major component of hemicellulosic polysaccharides, which have a backbone formed by *β*-1,4-linked D-glucose that are regularly substituted with branches of *α*-1,6-xylose (2). In addition, xylosyl residues can be further modified by *β*-1,2-connected α-L-arabinose or galactose (3). Xyloglucan exists as a storage carbohydrate in various tree seeds, e.g.*, Sinapis alba*, *Copaifera langsdorffii,* and *Tamarindus indica* (4). Among these, tamarind xyloglucan, mainly composed of galactoxyloglucan, has received widespread attention (5). In addition, xyloglucan oligosaccharides, recognized as a type of prebiotic, exhibit biological activities, such as lowering blood sugar and blood lipids (6). Physical, chemical, and enzymatic methods can be employed to produce xyloglucan oligosaccharides (7). Notably, enzymatic decomposition is often preferred due to its low environmental impact, mild reaction conditions, and high efficiency (8).

Xyloglucanases (EC 3.2.1.151) play a key role in the hydrolysis of xyloglucans as they are capable of breaking down *β*-1,4-glycosidic bonds to yield xyloglucan oligosaccharides (9). According to the Carbohydrate-Active EnZymes (CAZymes) database,^1^ xyloglucanases belong to glycoside hydrolase (GH) families 74, 45, 44, 16, 12, 9, and 5. Due to their functional properties, GH12 xyloglucanases have been utilized in various fields, including feed, food, biofuel, and paper industries (10, 11). Moreover, xyloglucanases with catalytic activity are gaining worldwide attention for their biotechnological applications, particularly in the fields of bioenergy and bioprocessing related to the environment.

Until now, most xyloglucanases have been functionally identified in various microorganisms, including *Paenibacillus* sp. S09 (3), *Rhizomucor miehei* CAU432 (11), and *Thermomonospora* sp. (7). These microbial xyloglucanases could be advantageous for industrial applications, particularly for producing xyloglucan oligosaccharides (12). *Trichoderma reesei* is currently the primary industrial source of commercial cellulases (13). *Trichoderma asperellum* is generally used as a biological control agent (14). Moreover, it has been shown to be an excellent candidate for lignocellulose hydrolysis, displaying robust growth, strong spore production, and rapid secretion of biomass-degrading enzymes, especially hemicellulases (15). However, the structure–function relationship and action model of acidophilic xyloglucanases from *T. asperellum* are rarely reported. Notably, enzymatic degradation for biomass transformation remains one of the most expensive technological processes (16). *Komagataella phaffii* is an excellent expression host for the efficient production of heterologous proteins due to its many advantages over *Escherichia coli*, particularly its minimal secretion of self-proteins (17). Hence, exploring the superior xyloglucanases will facilitate the production of xyloglucan oligosaccharides.

In this study, an acidophilic xyloglucanase (TaXEG12) belonging to GH12 was identified from *T. asperellum* ND-1, achieving an overexpression in *K. phaffii*, and biochemical characterization was investigated. Key active residues of TaXEG12 were explored through site-directed mutagenesis. Additionally, degradation models of TaXEG12 acting on tamarind xyloglucan were further analyzed. These results lay a theoretical foundation for understanding the TaXEG12 catalytic mechanism and developing TaXEG12 inhibitors.

## 2 Materials and methods

### 2.1 Materials

*T. asperellum* ND-1 (GenBank no: MH496612) was isolated from soil samples at Chifeng (Inner Mongolia, China). *Lactobacillus bulgaricus* (CICC accession number: 21101) was purchased from the China Center of Industrial Culture Collection. *K. phaffii* X-33 and the pPICZαA vector were obtained from Invitrogen (San Diego, CA, USA). Tamarind xyloglucan, beechwood xylan, and barley *β*-glucan were obtained from Megazyme (Wicklow, Ireland). Fructooligosaccharides (FOS) and sodium carboxymethyl cellulose (CMC-Na), sodium alginate, *p*-nitrophenyl-*α*-L-arabinofuranoside (pNPAf), pNP-*β*-d-galactopyranoside (pNPG), and commercial cellulase (TrCel) from *Trichoderma reesei* (ATCC 26921) were obtained from Sigma-Aldrich (USA).

### 2.2 Cloning and sequence analysis of xyloglucanase gene TaXEG12

The full-length *TaXEG12* gene was found in the genome of *T. asperellum* ND-1 (GenBank accession No. 2822840; 741 bp). The signal peptide of TaXEG12 was analyzed using the SignalP server.^2^ Total RNA extraction and synthesis of cDNA were performed using the RNeasy Plant Mini Kit (Qiagen, Crawley, UK) following the manufacturer’s instructions. The mature *TaXEG12* gene (*TaXEG12*-wt) was obtained from the cDNA of *T. asperellum* ND-1 by polymerase chain reaction (PCR) using the primers TaXEG12-F and TaXEG12-R (Supplementary Table S1). Additionally, codon optimization of the *TaXEG12* gene was carried out by ZixiBio Tech Co. (Nanjing) based on the preferred codon usage (average GC content of 41%) of *K. phaffii* and termed as *TaXEG12*-opt. *TaXEG12*-opt and *TaXEG12*-wt genes were, respectively, connected with the pPICZαA vector and subsequently identified by DNA sequencing.

Multiple sequence alignment of *TaXEG12* (GenBank accession No. XAM95724) with other GH12 xyloglucanases was achieved using Clustal X2 and then submitted to ESPript 3. Homology modeling and the catalytic region of TaXEG12 were investigated using SWISS-MODEL^3^ and Pfam,^4^ respectively.

### 2.3 Heterologous expression of TaXEG12 in *Komagataella phaffii*

The recombinant vectors containing *TaXEG12*-wt and *TaXEG12*-opt genes were designated as pPICZα-*TaXEG12*-wt and pPICZα-*TaXEG12-*opt, respectively. The constructed plasmids were linearized with *Sac*I, electroporated into *K. phaffii,* and preliminarily selected according to the manufacturer’s protocol (Invitrogen). The primers AOX-F and AOX-R (Supplementary Table S1) were used to further confirm the identities of the recombinant strains TaXEG12-wt and TaXEG12-opt.

Correctly constructed transformants of TaXEG12-wt and TaXEG12-opt were first inoculated into 5 mL yeast peptone dextrose (YPD) (28 °C, 12 h, 200 rpm). Enzyme induction was carried out in buffered methanol-complex medium (BMMY), as previously described (18). After 6 days of induction, enzyme concentrations were determined by the Bradford method (19), and expression levels were analyzed by sodium dodecyl sulfate-polyacrylamide gel electrophoresis (SDS-PAGE).

### 2.4 Enzyme activity assays

The xyloglucanase activity of TaXEG12 was assayed using the 3,5-dinitrosalicylic acid (DNS) method (20). In brief, the reaction mixture was prepared by combining 50 μL of diluted enzyme (50 mM sodium citrate buffer, pH 4.0) with 150 μL of a 5 mg/mL tamarind xyloglucan solution, resulting in a total volume of 300 μL. Then, the mixture was treated for 10 min at 40 °C, and the yield of reducing sugars was tested at 540 nm. One unit was defined as the amount of enzyme that produces 1 μmol of glucose equivalent per minute.

### 2.5 Biochemical characterization of TaXEG12

The effect of temperature and pH on the xyloglucanase activity of TaXEG12 was evaluated as previously described (18). The optimal pH of TaXEG12 was assayed at 50 °C in 50 mM buffer solutions (pH 2–8). Additionally, pH stability was determined by evaluating residual activity after pre-incubation in different buffer solutions at 25 °C for 1 h.

Enzyme activities were detected at varying temperatures (20–80 °C) to determine the optimal temperature for TaXEG12. Thermostability was evaluated by testing residual activity after pre-incubation of TaXEG12 at 20–80 °C for 1 h.

To observe the effects of metal ions (Zn^2+^, Cu^2+^, Ba^2+^, Mn^2+^, Al^3+^, Li^+^, Fe^3+^, K^+^, Cd^2+^, Ca^2+^, Pb^2+^, NH_4_^+^, Fe^2+^, Ni^2+^, Co^2+^, and Mg^2+^) and chemicals (SDS, urea, and EDTA), TaXEG12 was pre-incubated with 10 mM and 2 mM concentrations of these reagents for 1 h under optimal pH conditions, and its residual activity was subsequently tested. The mixture without additives served as the control (100%). The effects of different concentrations of 0–3.42 M ethanol and 0–20% (v/v) NaCl on TaXEG12 activity were also analyzed as described above.

### 2.6 Site-directed mutagenesis and computational analysis

Homology modeling of TaXEG12 was conducted using the SWISS-MODEL server, with GH12 xyloglucanase (PDB id: 4npr.1) from *Aspergillus niveus* serving as a template. The predicted ribbon diagram and molecular surface of TaXEG12 were analyzed using PyMol software. To explore the interactions between TaXEG12 and the substrate, xyloglucan was docked into the homology model using the LibDock program. Based on the optimized binding free energy and the simulated 3D structure, several residues (Asp^122^, Asp^126^, Asp^181^, Glu^94^, Glu^140^, and Glu^227^) were predicted to be crucial sites and were identified by site-directed mutagenesis. Briefly, these residues were all mutated to alanine using the Fast Mutagenesis Kit V2 (Vazyme Biotech Co., Ltd), with pPICZ*α*-*TaXEG12-*opt serving as the template. Corresponding primers for the mutations were designed and synthesized (Supplementary Table S1).

### 2.7 Substrate specificity

The substrate specificity of TaXEG12 was measured using 5 mM *p*-nitrophenyl-α-L-arabinofuranoside (pNPAf) and pNP-*β*-d-galactopyranoside (pNPG) as described previously (18). Briefly, 100 μL of diluted enzyme was mixed with 100 μL of pNPAf or pNPG and pretreated at 40 °C for 10 min. The released pNP was tested at 405 nm. For barley β-glucan, CMC-Na, beechwood xylan, sodium alginate, and locust bean gum, the enzyme activity of TaXEG12 was quantified using the DNS method (20).

### 2.8 High cell density fermentation

High cell density fermentation of the constructed strain TaXEG12-opt was performed in a 7-L fermentor at 29 °C (Bio Flo 115, NBS, USA) as described previously (11, 16). Samples were collected every 8 h to analyze xyloglucanase activity and cell concentration (OD_600_). Enzyme concentrations were determined using the Bradford method, and expression levels were analyzed by SDS-PAGE.

### 2.9 Analysis of the hydrolysis products and prebiotic activity

TaXEG12 (30 U) was incubated (reaction volume 5 mL) with 10 mg/mL of tamarind xyloglucan at 40 °C under optimal pH conditions. Enzyme-free samples were used as controls. The reactions were terminated by boiling for 3 min and detected using TLC or ESI-MS.

Hydrolysis products were developed on silica gel plates (cat # 60F 254; Merck; Darmstadt, Germany) using a solvent mixture of n-butyl alcohol/acetic acid/water (2,1,1, v/v). After being incubated with 5% sulfuric acid, the products were revealed by heating for 5 min at 100 °C.

To further confirm the products, a 2 μL sample was detected using the UPLC1290-6540B Q-TOF (Agilent Co., Santa Clara, CA, USA) in positive-ion mode with the following settings: sheath gas at 30 AU; nebulizer at 0.3 bar; capillary voltage at 3.0 kV; mass range from 20 to 2000 m/z; and dry gas flow rate at 13 L/min. The resulting fragment spectra were compared with previously published spectra of sodium alginate degradation products (21, 22).

To evaluate prebiotic activity, the hydrolysis products (0–3.0%, v/v) were mixed with de Man Rogosa Sharpe (MRS) agar as described previously (23). Then, *Lactobacillus bulgaricus* was cultured at 36 °C for 72 h. The number of *L. bulgaricus* colonies was analyzed.

### 2.10 Synergistic action

Lignocellulosic biomass (corn bran and apple pulp) was pretreated as described previously (24). The enzymatic hydrolysis of 5% (w/v) xyloglucan-containing substrates (corn bran or apple pulp) by TaXEG12 (85 U/g biomass) or TrCel (18 U/g biomass) simultaneously or sequentially was carried out at 50 °C and pH 5.0, mixing at 200 rpm for 24 h. In the sequential experiment, the first enzyme was interrupted by boiling for 5 min after hydrolysis for 24 h. Then, another enzyme was loaded, and the reaction was stopped by boiling for 5 min after hydrolysis for 24 h under the same conditions. Reactions containing substrate alone were used as blank controls.

### 2.11 Statistical analysis

Duncan’s tests (SPSS 20) and one-way analysis of variance (ANOVA) were used for statistical analysis. A significance level of *p* ≤ 0.05 was established.

## 3 Results and discussion

### 3.1 Sequence analysis of *the TaXEG12* gene

The xyloglucanase gene *TaXEG12* (Gene ID: 2822840) amplified from the *T. asperellum* ND-1 genome was 741 bp, containing 246 amino acids with an 18-residue predicted signal peptide (MKFIHLVSAFFTANTAAA). The mature TaXEG12 had a calculated isoelectric point (pI) of 8.1 and a molecular mass of 24.5 kDa. Sequence alignment suggested that TaXEG12 displayed 46.8% similarity with a GH12 xyloglucan-specific endo-*β*-1,4-glucanase from *A. niveus* (4npr) and 44.3% similarity with the already characterized GH12 xyloglucanase RmXEG12A from *R. miehei* CAU432 (JQ901459).

**Figure 1:**
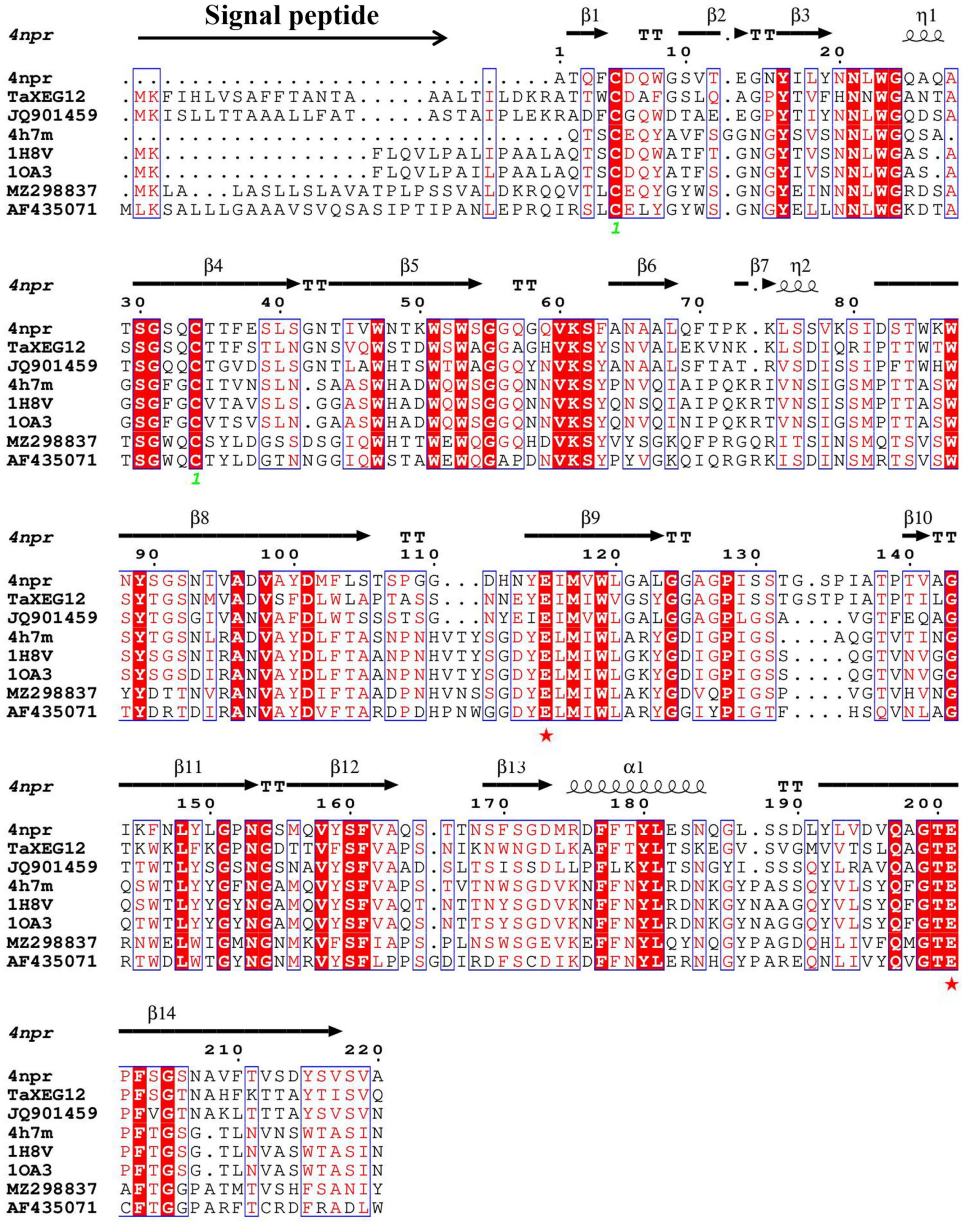
Multiple sequence alignment of TaXEG12 (GenBank accession No. XAM95724) with other GH12 xyloglucanases, including *Aspergillus niveus* (PDB id: 4npr), *Rhizomucor miehei* CAU432 (JQ901459), *Trichoderma harzianum* (4h7m), *T. reesei* (1H8V), *Hypocrea schweinitzii* (1OA3), *Chaetomium* sp. CQ31 (MZ298837), and *Humicola grisea* (AF435071). The two catalytic residues (E140 and E227) are marked using red asterisks. The alignment was performed using ClustalX2 and ESPript 3.0.

Two glutamic acid residues (E140, E227) were considerably conserved in β9 and β14 of these enzymes (Figure 1), suggesting that they may be responsible for the enzymes’ catalytic activity. Additionally, the GC content of the *TaXEG12* gene was 44.7%, and the codon adaptation index was 0.85 after codon optimization (Supplementary Figure S1).

### 3.2 Enhancement of TaXEG12 expression in *K. phaffii*

The similarity between the codon-optimized gene (*TaXEG12-*opt) and the native gene (*TaXEG12-*wt) was 77%, with 158 nucleotides being optimized (Supplementary Figure S1). The constructed transformants, TaXEG12-wt and TaXEG12-opt, were further confirmed by the PCR (Supplementary Figure S2).

**Figure 2:**
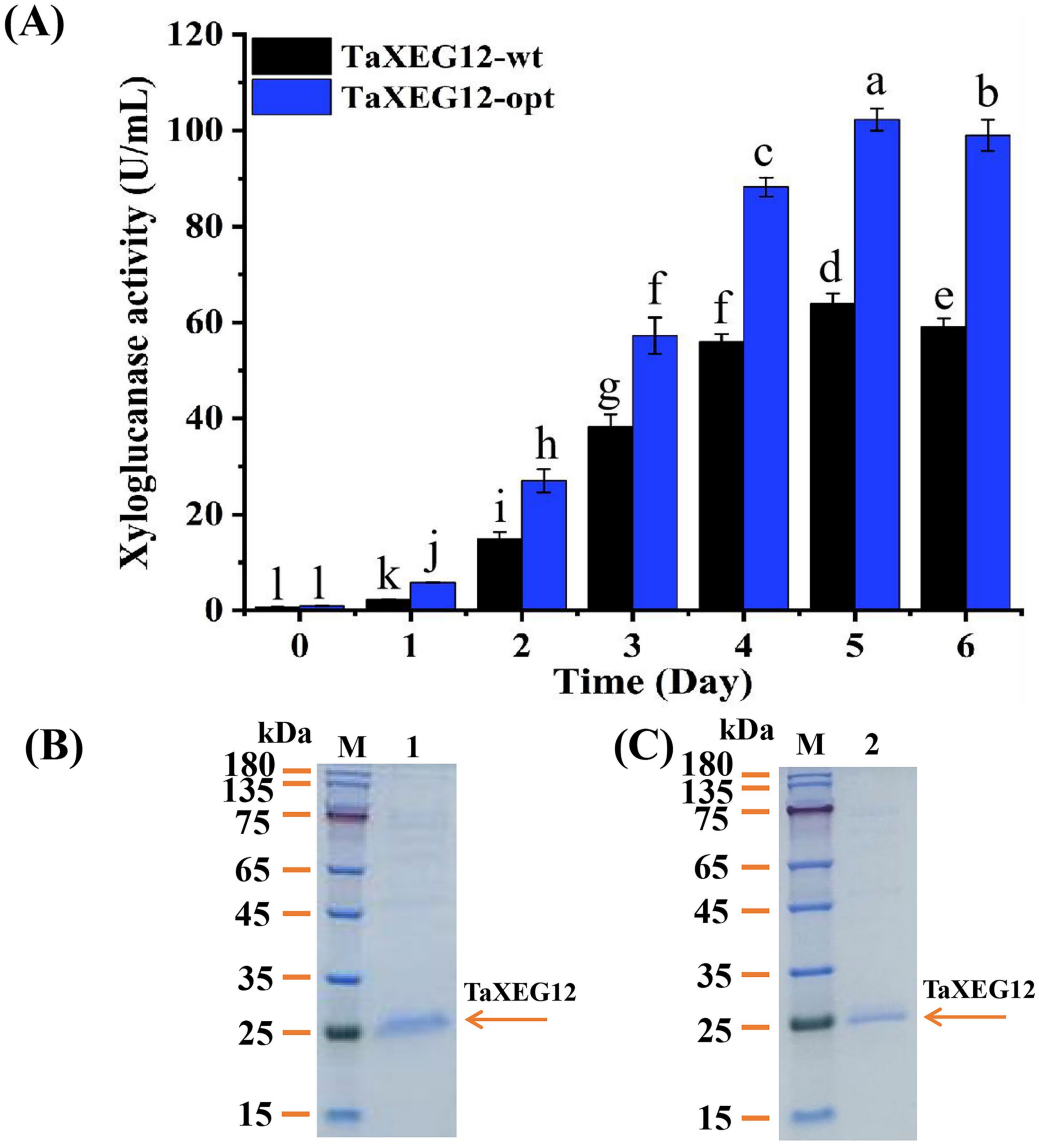
Optimized expression of TaXEG12 in *K. phaffii*. **(A)** Recombinant strains TaXEG12-wt and TaXEG12-opt (schematic). **(B)** Enzyme activity of TaXEG12 with tamarind xyloglucan as substrate. Error bars indicate the standard deviation of three biological replicates. Columns with different letters indicate a significant difference (*p <* 0.05). **(C)** Analysis of optimized TaXEG12 on 12% SDS-PAGE. Lane M: standard protein markers. Lanes 1: culture supernatant of TaXEG12-opt; Lanes 2: culture supernatant of TaXEG12-wt.

After a 144-h induction with 1% (v/v) methanol, the xyloglucanase activity of the constructed strain TaXEG12-opt increased to a maximum of 102.3 ± 2.3 U/mL at 120 h (Figure 2A), which was 1.6-fold higher than that of TaXEG12-wt (63.9 ± 2.2 U/mL) (Figure 2A). So far, many heterologous proteins have been overexpressed via codon optimization in *K. phaffii,* including endoglucanase rMt-egl from *Myceliophthora thermophila* BJA (25) and lipase from *Candida rugosa* (26). Additionally, *T. asperellum* secreted various CAZymes involved in the degradation of biomass polysaccharides, except for xyloglucan (15).

SDS-PAGE analysis of recombinant protein induced from either TaXEG12-opt or TaXEG12-wt revealed a major band with a molecular mass of approximately 25 kDa (Figure 2B), which matched with its theoretical value. The molecular weight of TaXEG12 was comparable to that of xyloglucanases PoxXEG12A (from *Penicillium oxalicum*, 26.5 kDa) (4) and RmXEG12A (from *R. miehei* CAU432, 21.9 kDa) (14).

### 3.3 The influence of pH and temperature on TaXEG12 activity

**Figure 3:**
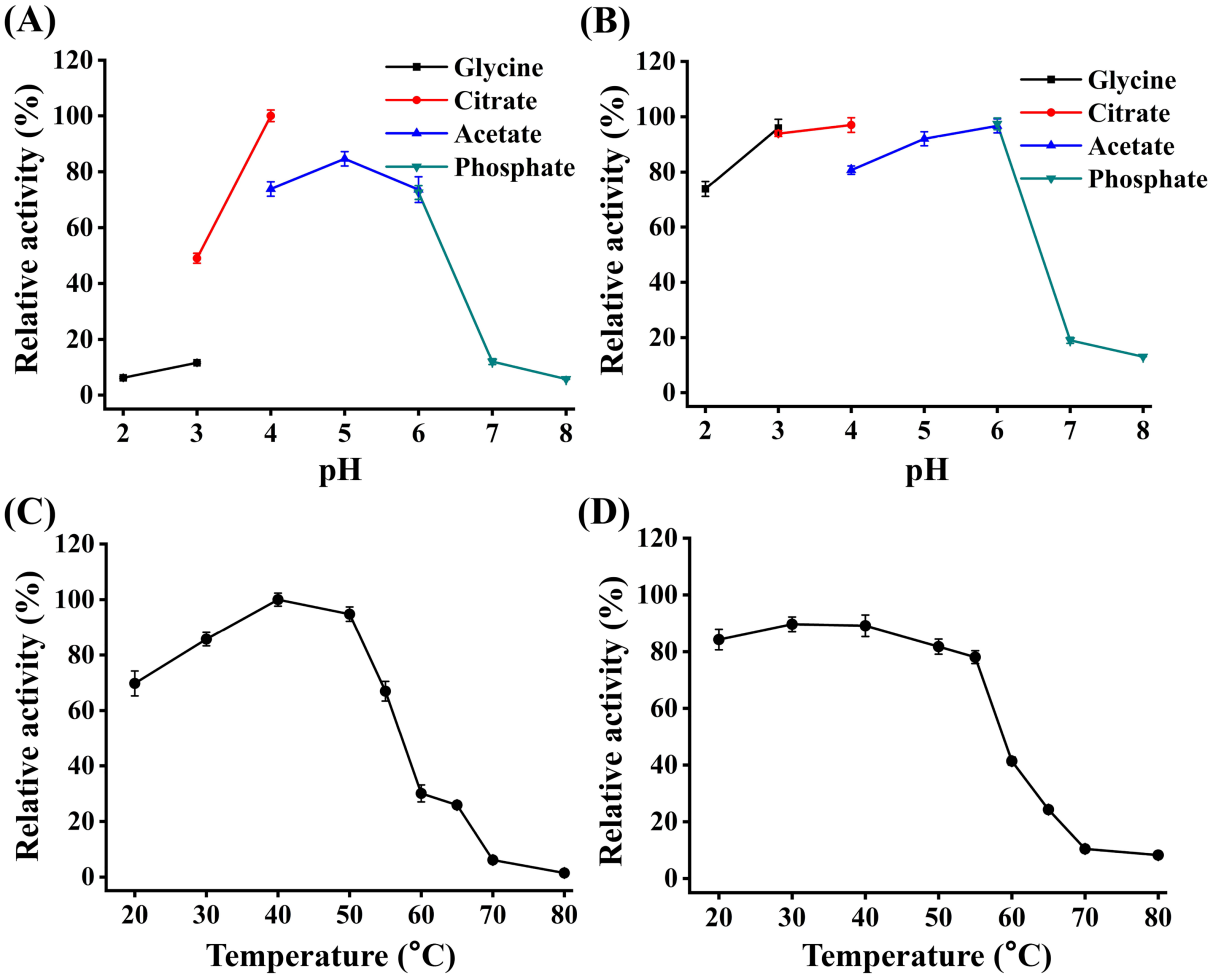
Effects of pH and temperature on TaXEG12 activity with tamarind xyloglucan as substrate. **(A)** Optimal pH. **(B)** pH stability. **(C)** Optimal temperature. **(D)** Thermostability. Values shown are mean ± SD from triplicate experiments.

TaXEG12 exhibited maximum activity at pH 4.0 and maintained over 40% peak activity at a pH of 3.0–6.0, suggesting that TaXEG12 is an acidophilic xyloglucanase (Figure 3A). When the pH exceeded 6, however, the activity decreased significantly, and only 5% peak activity was maintained at pH 8 (Figure 3A). Similar results have been characterized for some GH12 fungal xyloglucanases, such as SaGH74A from *Streptomyces avermitilis* NBRC14893 (pH 5.5) (27) and Xgh12B from *Aspergillus cervinus* (pH 5.0) (28). Moreover, TaXEG12 showed tolerance under acidic conditions, retaining more than 70, 90, and 80% of its activity at pH 2, 3, and 4, respectively (Figure 3B). However, it maintained approximately 19 and 13% activity at pH 7.0 and 8.0, respectively (Figure 3B).

With respect to optimal temperature, TaXEG12 appeared to have maximum activity at 40 °C and exhibited over 60% of the peak activity at 20–55 °C (Figure 3C). However, the xyloglucanase activity of TaXEG12 was rapidly and completely lost when incubated at 60 and 80 °C (Figure 3C). The results differed from those for xyloglucanase (RmXEG12A), as reported by Wang et al. (11), which appeared to have optimal activity at 65 °C and pH 7.0. Additionally, TaXEG12 was found to be stable at a temperature range of 20–50 °C for 1 h, maintaining over 80% activity (Figure 3D). The enzymes that display maximum activity at approximately 40 °C and pH 4.8 of the digestive tract environment may be suitable for feed fields (29, 30).

### 3.4 The inhibitory influence of metal ions and chemical reagents on TaXEG12 activity

As shown in Table 1, Mg^2+^ and Fe^2+^ improved the catalytic activity by 1.9–17.6% at both concentrations (2 and 10 mM). TaXEG12 activity increased to 113.74 ± 2.49% and 107.94 ± 3.75% in the presence of 2 mM of Ni^2+^ and Li^+^, respectively; however, at a concentration of 5 mM, these metal ions had only a slight effect on activity (Table 1). However, 2 mM Mn^2+^, Pb^2+^, and Cu^2+^ showed effect in inhibiting the activity to 39, 30, and 22%, respectively (Table 1). Similarly, significant inactivation by Cu^2+^ was also detected for xyloglucanases from *P. oxalicum* (63.9%) (4) and *A. cervinus* (50%) (28). Other listed metal ions (2 mM) showed little or no effect on TaXEG12 activity (Table 1). When the final concentration was enhanced to 10 mM, K^+^ and Al^3+^ marginally stimulated the enzyme activity (less than 7%), while TaXEG12 activity was significantly repressed by Mn^2+^ (24.99 ± 0.32%), Pb^2+^ (25.38 ± 0.32%), Cu^2+^ (25.20 ± 0.48%), and Fe^3+^ (75.07 ± 0.63%), respectively (Table 1). In addition, xyloglucanase activity of TaXEG12 was almost completely decreased by 2 and 10 mM SDS (less than 6%) (Table 1), which exhibited stronger inactivation than that of RmXEG12B (89.5%) from *R. miehei* (10) and rPgl5A (52.3%) from *Paenibacillus* sp. S09 (3).

**Table 1:** Inhibitory effects of metal ions and chemical reagents on TaXEG12 activity.

Chemicals	Relative activity* (%) (mean ± SD, *N =* 3)	
	2 mM	10 mM
Control	100 ± 3.36^d^	100 ± 1.06^d^
Mg^2+^	107.99 ± 1.01^b^	101.98 ± 4.78^d^
NH_4_^+^	104.71 ± 1.33^c^	97.22 ± 1.99^e^
Ni^2+^	113.74 ± 2.49^a^	99.27 ± 3.47^d^
Li^+^	107.94 ± 3.75^b^	99.74 ± 1.87^d^
Fe^2+^	107.78 ± 4.26^b^	117.56 ± 1.71^a^
K^+^	99.46 ± 1.64^d^	107.06 ± 2.34^b^
Cd^2+^	101.76 ± 0.86^d^	93.98 ± 1.17^f^
Zn^2+^	101.09 ± 1.15^d^	84.99 ± 2.66^h^
Al^3+^	95.24 ± 1.95^e^	104.86 ± 1.52^c^
Mn^2+^	61.20 ± 1.53^j^	24.99 ± 0.32^j^
Pb^2+^	70.72 ± 1.10^i^	25.38 ± 0.32^j^
Ca^2+^	93.81 ± 6.61^f^	89.23 ± 2.62^g^
Co^2+^	97.32 ± 1.52^e^	93.36 ± 0.39^f^
Ba^2+^	102.85 ± 1.95^c^	98.59 ± 3.87^e^
Cu^2+^	78.49 ± 2.71^h^	25.20 ± 0.48^j^
Fe^3+^	90.48 ± 1.09^g^	75.07 ± 0.63^i^
Urea	107.39 ± 6.19^b^	100.78 ± 3.59^d^
SDS	6.53 ± 0.20^k^	1.23 ± 0.04^k^
EDTA	115.28 ± 2.11^a^	95.97 ± 1.80^f^

*The activity in the absence of metal ions was considered as 100%. Data reflect the mean ± SD (*n =* 3). The Duncan’s test was conducted in columns. Different superscript letters indicate a significant difference (*p <* 0.05).

**Figure 4:**
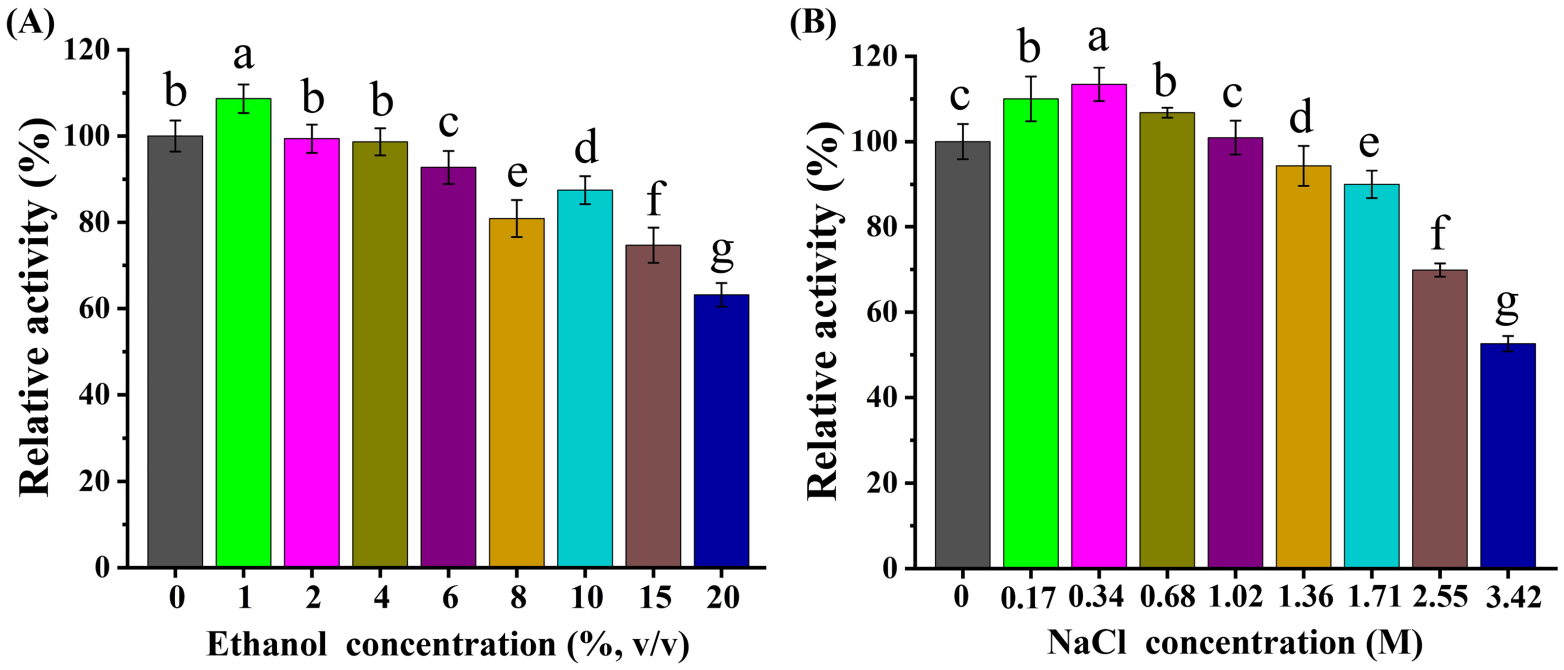
Effect of ethanol **(A)** and NaCl **(B)** concentration on TaXEG12 activity. Error bars indicate the standard deviation of three biological replicates. Columns with different letters indicate a significant difference (*p <* 0.05).

Additionally, TaXEG12 showed more than 80 and 60% activity at 0–10% (v/v) and 20% (v/v) ethanol concentrations, respectively (Figure 4A). Similarly, TaXEG12 retained over 90% of xyloglucanase activity in the presence of 0–1.71 M NaCl and exhibited a maximum activity (113.4%) at 0.34 M NaCl (Figure 4B). This level of salt tolerance is comparable to or exceeds that of rPgl5A (102%, 10 mM) from *Paenibacillus* sp. S09 (3), PoxXEG12A from *P. oxalicum* (82.6%, 5 mM) (4), and RmXEG12A (104.5%, 1 mM) from *R. miehei* (10).

### 3.5 Site-directed mutagenesis and substrate docking studies

**Figure 5:**
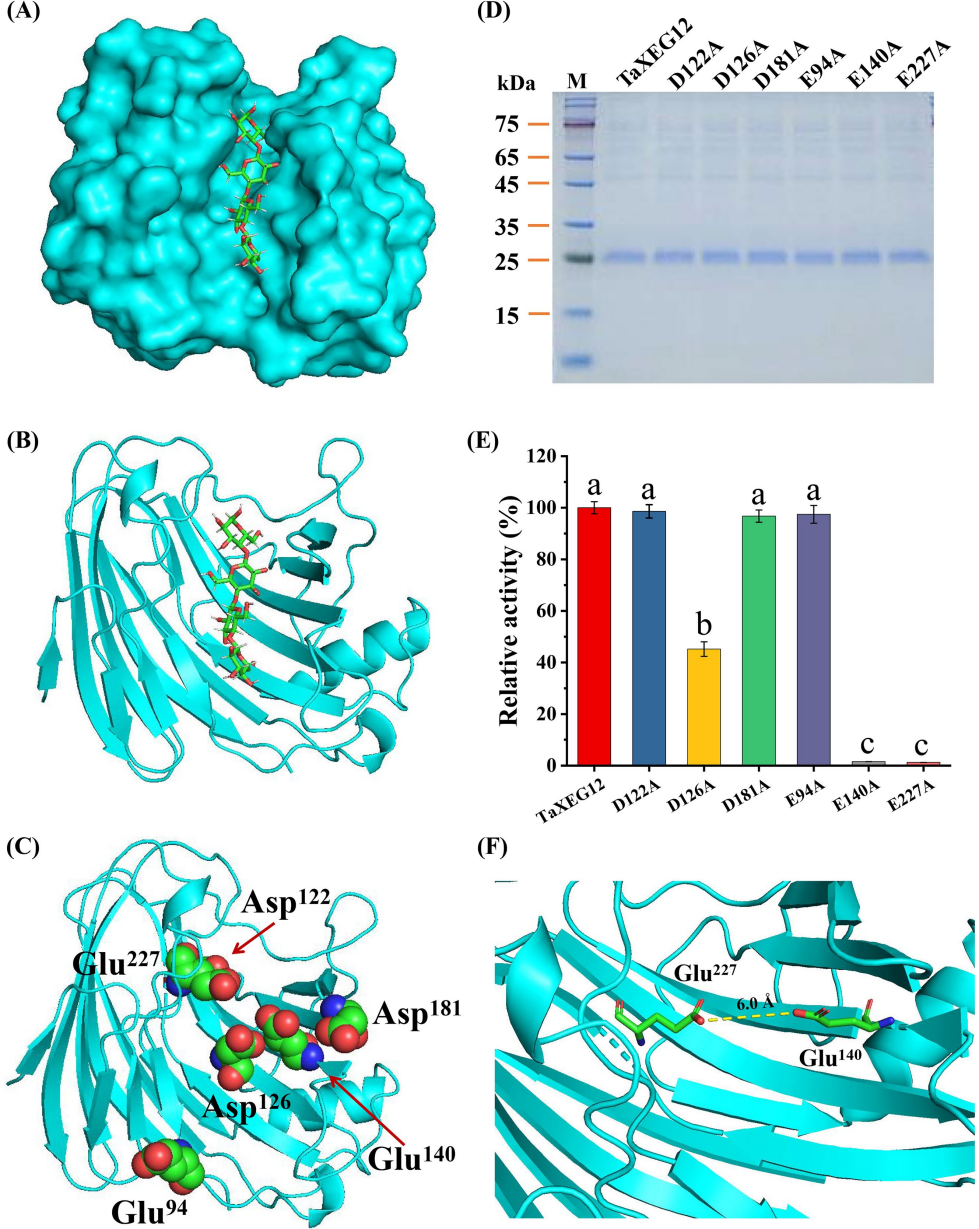
Identification of TaXEG5 active sites. The simulated molecular surface **(A)**, ribbon diagram **(B)**, and stereoview of the predicted active sites **(C)** of TaXEG5. SDS-PAGE analysis **(D)** and enzyme activities **(E)** of TaXEG5 and its mutants, with Tamarind xyloglucan as a substrate. Error bars indicate the standard deviation of three biological replicates. Columns with different letters indicate a significant difference (*p <* 0.05). **(F)** Enzyme structure of the catalytic center, showing distances (Å) between catalytic residues.

As shown in Figure 5A, computational modeling and docking studies revealed that the TaXEG12 structure had a substrate-binding cleft, which was similar to other glycoside hydrolases, such as endoglucanase from *Trichoderma harzianum* (31). The TaXEG12 catalytic cleft provided space for xyloglucan (Figure 5B). Two glutamic acid residues (E140, E227) were located in the catalytic cleft (Figure 5C).

The analysis of the optimized binding free energy and simulated 3D structure revealed that six residues (Asp^122^, Asp^126^, Asp^181^, Glu^94^, Glu^140^, and Glu^227^) were present in the catalytic center (Figure 5C). SDS-PAGE analysis displayed that the molecular weights of TaXEG12 and its mutants were approximately 25 kDa (Figure 5D). The substitution of Glu^140^ and Glu^227^ with Ala could result in a dramatic reduction of TaXEG12 activity, retaining 1.5 and 1.2%, respectively (Figure 5E). The mutants D122A, D181A, and E94A exhibited little or no effect on TaXEG12 activity (Figure 5E), but D126A decreased its activity by 55% (Figure 5E), suggesting that Asp^126^ played an auxiliary role in the degradation of tamarind xyloglucan. Moreover, the distance of the crucial catalytic sites Glu^140^-Glu^227^ for TaXEG12 is approximately 6.0 Å (Figure 5F), which is similar to the distance between the corresponding residues (Glu^133^-Glu^219^) in the 4npr.1 structure (5.6 Å).

The Glu sites were usually recognized as acid/base residues and engaged in key hydrolysis reactions with substrates (32–35). Similarly, Glu^119^ and Glu^205^ and Glu^155^ and Glu^243^ were reported as the active sites for GH12 xyloglucanases from *A. aculeatus* (36) and *Bacillus licheniformis* (33), respectively.

### 3.6 Substrate specificity of TaXEG12

TaXEG12 exhibited the maximum catalytic ability toward tamarind xyloglucan (102.3 ± 2.3 U/mL) and much lower activity toward barley *β*-glucan (12.7 ± 0.5 U/mL) (Table 2). Moreover, little or no detectable catalytic capacity was observed for pNPG, pNPAf, and other polysaccharides (Table 2), suggesting their strict substrate specificity.

**Table 2:** Hydrolysis of different substrates catalyzed by TaXEG12.

Substrate	Concentration [1% (w/v)]	Activity of TaXEG12 (U/mL) (mean ± SD; *n =* 3)
Tamarind xyloglucan	1	102.3 ± 2.34
Barley β-glucan	1	12.7 ± 0.45
Sodium carboxymethyl cellulose (CMC-Na)	1	0.7 ± 0.05
Beechwood xylan	1	0.5 ± 0.08
Sodium alginate	1	0.0 ± 0.00
Locust bean gum	1	0.0 ± 0.00
*p*-Nitrophenyl-α-L-arabinofuranoside (pNPAf)	5 mM	0.0 ± 0.00
*p*-Nitrophenyl-β-D-galactopyranoside (pNPG)	5 mM	0.0 ± 0.00

### 3.7 Bioreactor scale-up of TaXEG12

**Figure 6:**
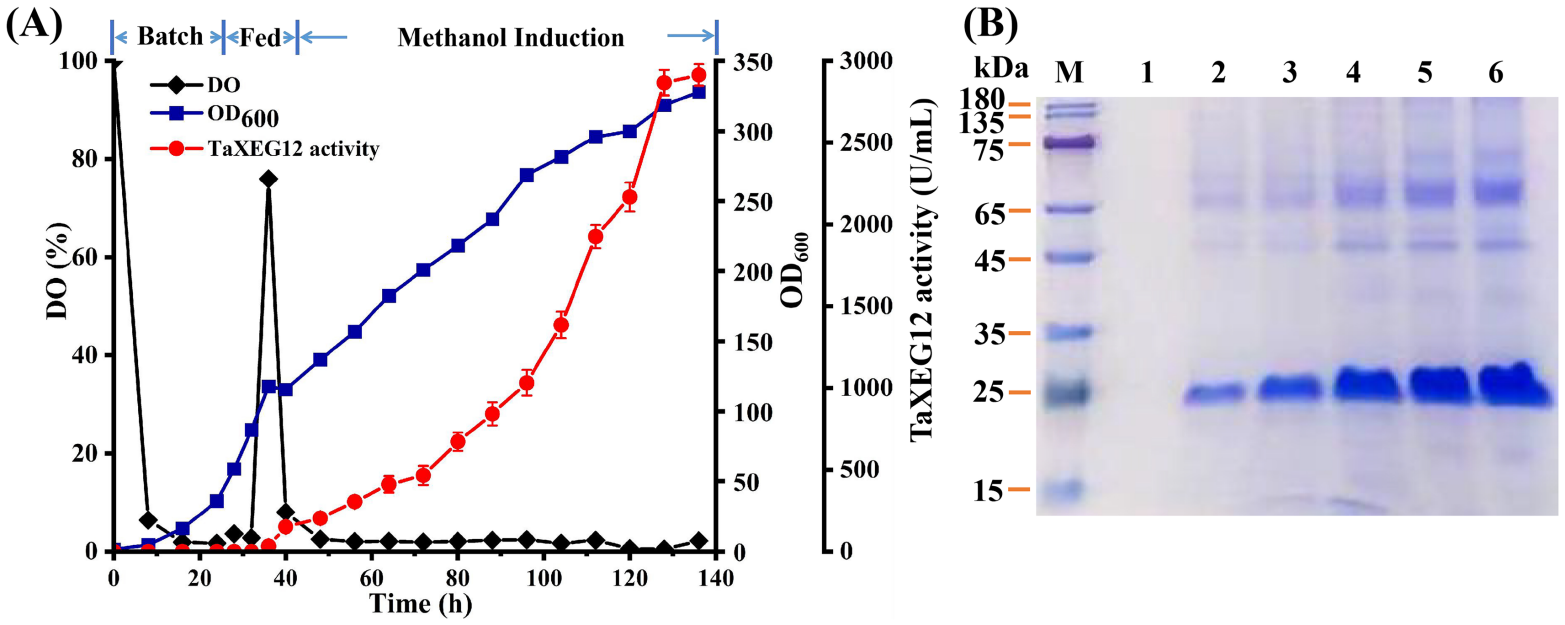
Fed-batch fermentation **(A)** and SDS-PAGE **(B)** analysis of the constructed strain TaXEG12-opt in a 7-L bioreactor. TaXEG12 activity (●), OD_600_ (◼), DO (◆). Lanes: M, standard protein molecular weight markers; 1, 32 h; 2, 56 h; 3, 80 h; 4, 104 h; 5, 128 h; and 6, 136 h.

To further enhance the xyloglucanase activity, TaXEG12-opt was selected for fed-batch fermentation in a 7-L bioreactor. During fermentation, glycerol feeding was initiated at 32 h and continued for 8 h (Figure 6A). After 136 h of fermentation, the volumetric productivity of TaXEG12 reached 107150.7 ± 2415.4 U/(mL·h)‌. Moreover, the specific activity and xyloglucanase activity of TaXEG12 reached 3643.1 ± 82.1 U/mg and 2914.5 ± 65.7 U/mL, respectively (Figure 6A); the level was 28.5-fold higher than that obtained in a shake flask (102.3 ± 2.3 U/mL). Moreover, the induction result of TaXEG12 was higher than that of xyloglucanase from *Penicillium oxalicum* (1.5 U/mL) (4) and *Aspergillus cervinus* (1,000 U/mL) (28). Meanwhile, cell concentration (OD_600_) improved to a maximum of 328 (Figure 6A). SDS-PAGE was used to analyze the fermentation supernatant during the induction process. Similar to flask culture, a dominant protein band of 25 kDa was detected (Figure 6B).

### 3.8 Analysis of hydrolysis products and prebiotic activity

Hemicellulose typically consists of 20–25% of the dry weight of lignocellulosic biomass, while mixed-linkage glucans and xyloglucans serve as the main components of hemicelluloses in crop wastes (2). Enzymatic degradation of hemicelluloses can stimulate cellulose decomposition by improving cellulase affinity and producing various value-added products, such as prebiotic oligosaccharides derived from mixed-linkage *β*-glucans and xyloglucans (37).

**Figure 7:**
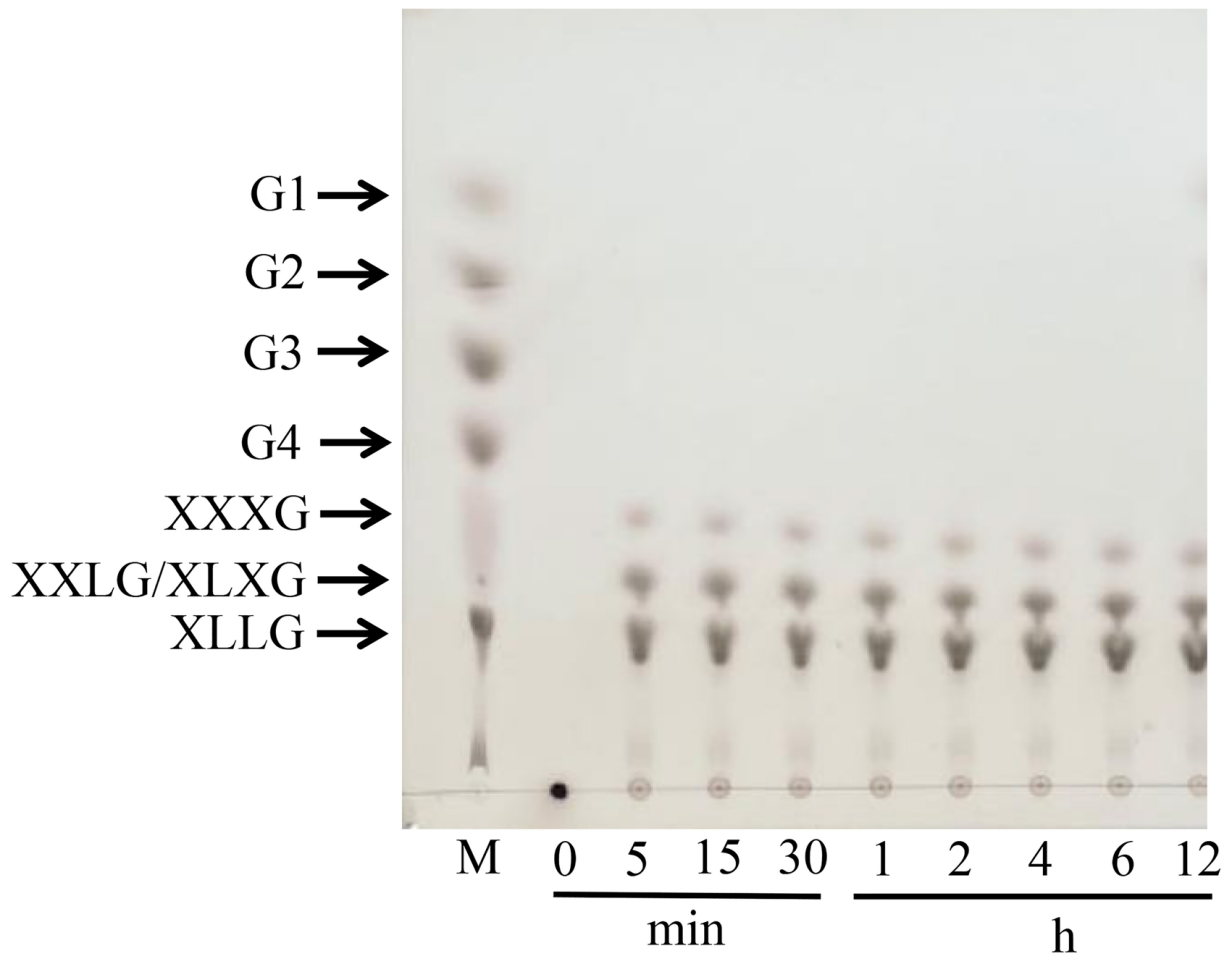
Time-course hydrolysis of 10 mg/mL tamarind xyloglucan by TaXEG12. Mixture of xyloglucan oligosaccharides (XXXG, XLXG/XXLG, and XLLG), cellotetraose (G4), cellotriose (G3), cellobiose (G2), and glucose (G1) was used as a standard. G, unbranched glucosyl residues; X, xylosyl-(1,6)-glucosyl residues; L, galactosyl-(1,2)-xylosyl-(1,6)-glucosyl residues.

**Figure 8:**
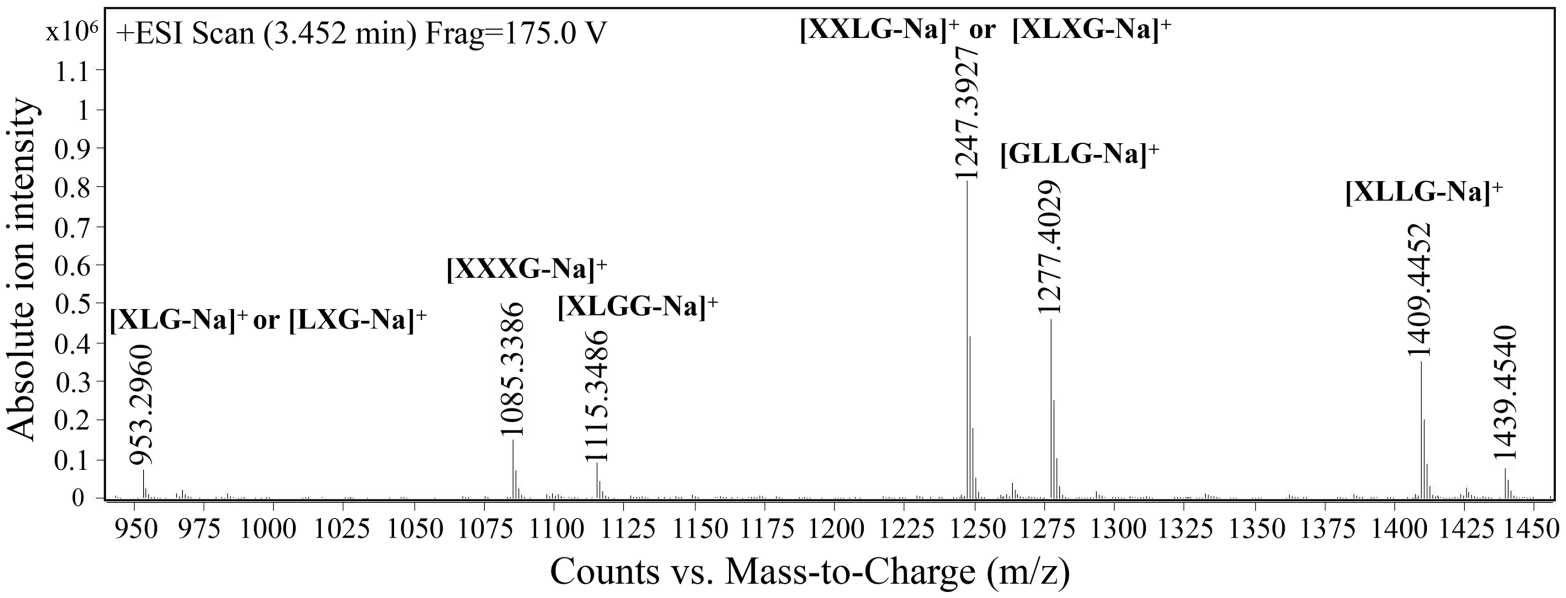
Analysis of the degradation products of tamarind xyloglucan after incubation with TaXEG12 at pH 4.0 and 40 °C for 24 h. The released oligosaccharides were separated and analyzed by ESI-MS. G, unbranched glucosyl residues; X, xylosyl-(1,6)-glucosyl residues; L, galactosyl-(1,2)-xylosyl-(1,6)-glucosyl residues.

As shown in Figure 7, XLLG, XXLG/XLXG, and XXXG served as the main hydrolytic products degraded from tamarind xyloglucan by TaXEG12. After 12 h of hydrolysis, the fragments with m/z 1,409, 1,247, and 1,085 corresponding to the [M + Na]^+^ adduct ions of XLLG, XXLG/XLXG, and XXXG were also detected using the ESI-MS (Figure 8). Similar phenomena have been reported for some xyloglucanases, such as RmXEG12A from *R. miehei* CAU432 (11), Xgh12B from *A. cervinus* (28), and Sco6545 from *Streptomyces coelicolor* A3 (2, 38). However, the xyloglucanase XEG74 from *Paenibacillus* sp. strain KM21 could hydrolyze not only after glucose residues but also after xylose motifs, producing XXX, XXXG, and GXXXG (39). In addition, the activity of TaXEG12 produced three reduced oligosaccharide ions: LXG/XLG (m/z 953), XLGG (m/z 1,115), and GLLG (m/z 1,277) (Figure 8). This suggests that TaXEG12 acts as endo-lytic, cleaving at unsubstituted glucose residues (21).

**Figure 9:**
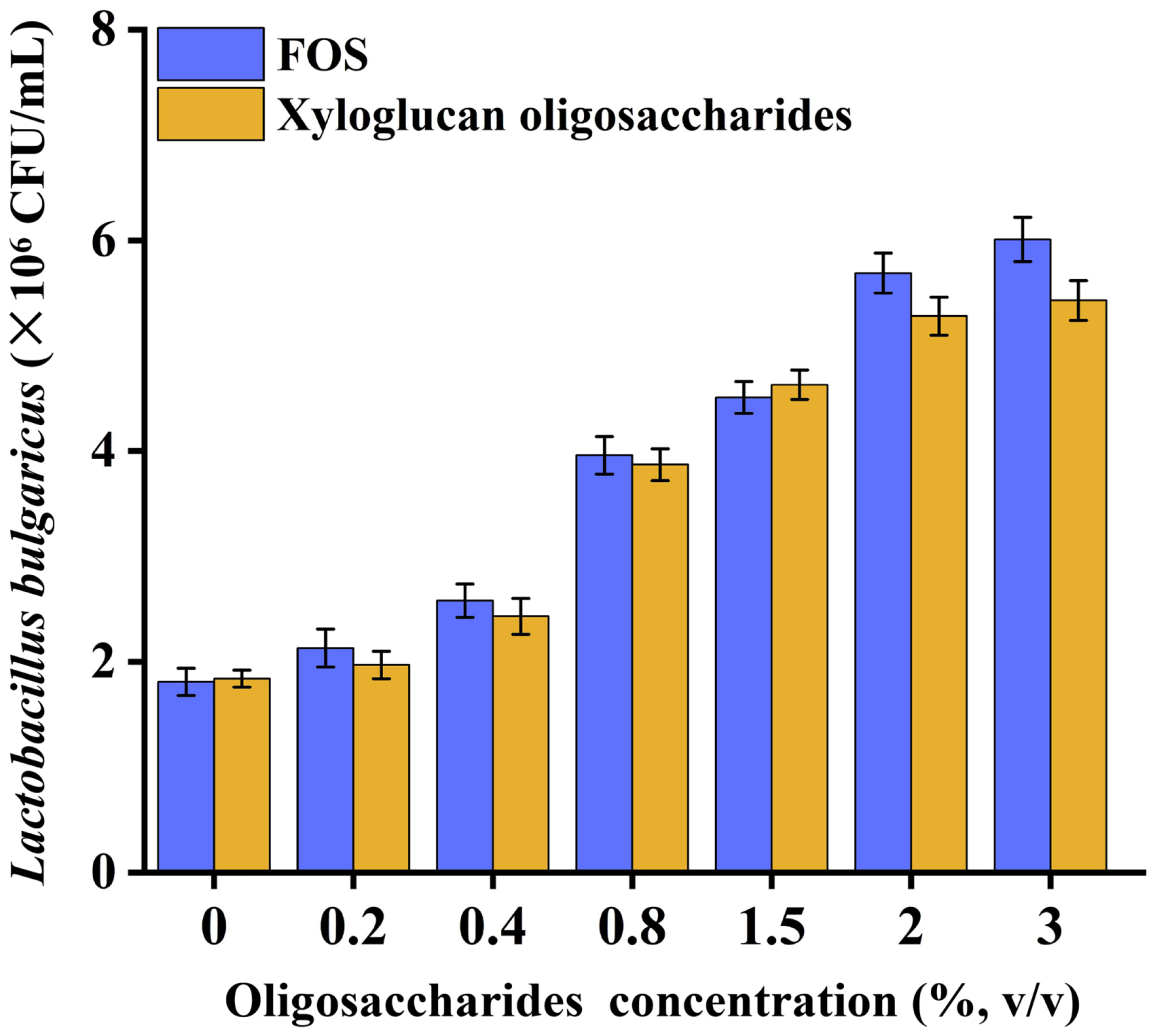
Effect of fructooligosaccharides (FOS) and xyloglucan oligosaccharides on the growth of *Lactobacillus bulgaricus*.

Prebiotics are non-digestible food or feed additives that have been proven to facilitate the proliferation of beneficial microorganisms (40). Common prebiotics, including inulin, fructooligosaccharides (FOS), and xylooligosaccharides (XOS), can effectively improve the proportion of probiotics (*Lactobacillus* and *Bifidobacterium*) and enhance the short-chain fatty acid production (41, 42). As a key prebiotic, xyloglucan oligosaccharides are garnering great attention due to their benefits for human health (6). As shown in Figure 9, the addition of xyloglucan oligosaccharides (1.5%) promoted the growth of *L. bulgaricus* by 2.5-fold, which is similar to the effect seen with the xyloglucanase RmXEG12A from *R. miehei* CAU432 (11). Compared to the control, FOS and xyloglucan oligosaccharides (2.0%, w/w) promoted the growth of *L. bulgaricus* by 3.1- and 2.8-fold, respectively (Figure 9).

### 3.9 Synergistic effect

**Figure 10:**
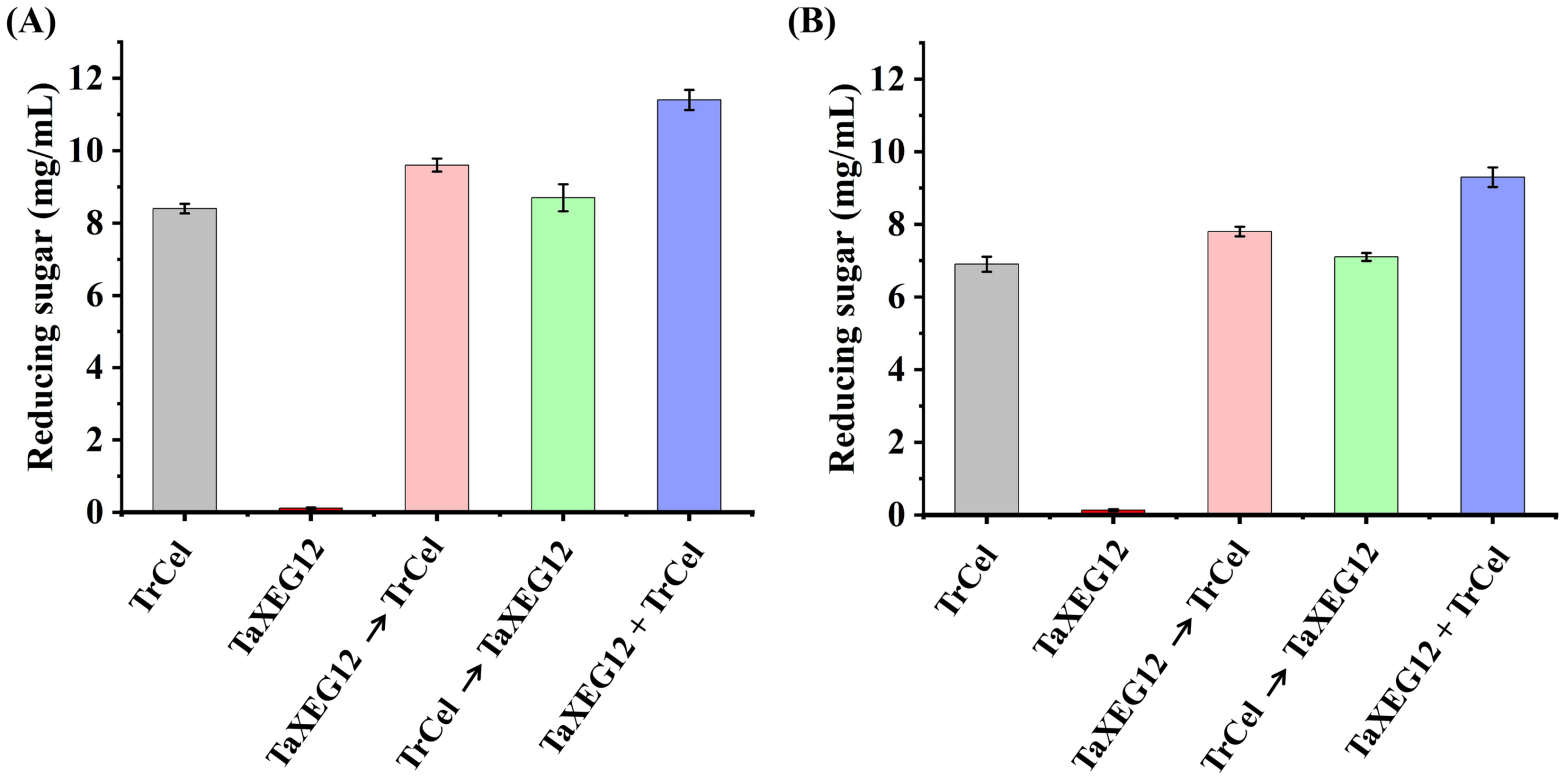
Synergistic action of TaXEG12 and commercial cellulase TrCel in biomass hydrolysis. **(A)** corn bran and **(B)** apple pulp. Error bars indicate the standard deviation of three biological replicates.

Corn bran and apple pulp are abundant and cost-effective resources for the production of high-value derivatives (43, 44). Xyloglucan, the major component of hemicellulose, is closely associated with cellulose and greatly decreases the catalytic efficiency of cellulase (45). Thus, the decomposition and conversion of xyloglucan-containing biomass (corn bran or apple pulp) require the cooperative interactions of different biomass-degrading enzymes, particularly cellulase and xyloglucanase (46). As shown in Figure 10, the simultaneous addition of TaXEG12 and TrCel resulted in higher sugar production compared to the addition of TaXEG12 or TrCel alone. The synergy degrees of corn bran and apple pulp by TaXEG12 and TrCel degradation were 1.34 (Figure 10A) and 1.32 (Figure 10B), respectively. These cooperative results suggest that TaXEG12 is capable of acting synergistically with cellulase. This phenomenon is similar to other described xyloglucanases, such as AbiXeg12a from *Abortiporus biennis* (30) and *Tt*GH74 from *Thielavia terrestris* (46).

Additionally, the effects of the sequential addition of TaXEG12 and TrCel on the degradation of corn bran and apple pulp were further studied (Figure 10). The sequential decomposition (TrCel → TaXEG12) for corn bran (9.6 mg/mL) and apple pulp (7.8 mg/mL) displayed a slightly higher reducing sugar yields compared to the reverse order of the addition (TaXEG12 → TrCel). This reveals that the degradation activity of cellulases resulted in the generation of more catalytic sites for TaXEG12.

## 4 Conclusion

In this study, a GH12 xyloglucanase (TaXEG12) was identified from *T. asperellum* ND-1 and overexpressed in *K. phaffii* after codon optimization. TaXEG12 exhibited ethanol tolerance, acidophilicity, and halophilicity. Glu^140^ and Glu^227^ are essential catalytic residues, while Asp^126^ plays auxiliary roles in xyloglucan degradation. Additionally, TaXEG12 possesses strict substrate specificity toward tamarind xyloglucan. TLC and ESI-MS analyses revealed that TaXEG12 hydrolyzed xyloglucan into xyloglucan oligosaccharides. These xyloglucan oligosaccharides could promote the growth of *L. bulgaricus*. In addition, TaXEG12 could enhance the action of commercial cellulase on corn bran and apple pulp. All of these preferable characteristics, particularly acidophilic ones, make TaXEG12 a promising candidate for preparing value-added xyloglucan oligosaccharides and cellooligosaccharides. Furthermore, crystallographic structure determination and analysis are needed to elucidate the interactions between TaXEG12 and tamarind xyloglucan.

## Data Availability

The datasets presented in this study can be found in online repositories. The names of the repository/repositories and accession number(s) can be found in the article/Supplementary material.

## Footnotes

1
http://www.cazy.org/

2
http://www.cbs.dtu.dk/services/signalP

3
https://swissmodel.expasy.org

4
http://pfam.xfam.org/
