# Supplementary material for: An acidophilic GH12 xyloglucanase from Trichoderma asperellum produces prebiotic oligosaccharides that promote probiotic growth
Source: Front Nutr. 2026 Jun 29;13:1850050. doi: 10.3389/fnut.2026.1850050 (PMC13357436; doi:10.3389/fnut.2026.1850050)
Supplement: Supplementary file 1 [file Table_1.doc]

**An acidophilic GH12 xyloglucanase from *Trichoderma asperellum* produces prebiotic oligosaccharides that promote probiotic growth**

**Table S1.** Oligonucleotide primers used in this study.

| **Primers** | **Sequence ( 5´- 3´ )** |
| --- | --- |
| TaXEG12-F  TaXEG12-R | GAATTCTTAACAATTCTCGACAAGCGG  TCTAGATTATTGAACA GAAATAGT |
| AOX-F | GACTGGTTCCAATTGACAAGC |
| AOX-R | GCAAATGGCATTCTGACATCC |
| **TaXEG12 mutants** | |
| D122A-F | GCTGCTGTCTCTTTCGATTTGTGGCTGGCACCAACTGC |
| D122A-R | CGAAAGAGACAGCAGCAACCATGTTAGAACCAGTGTAAGACCAGG |
| D126A-F | CTTTCGCTTTGTGGCTGGCACCAACTGCTTCTTCCAACAACGAG |
| D126A-R | GCCAGCCACAAAGCGAAAGAGACATCAGCAACCATGTTAGAACCAG |
| D181A-F | GGTGCTACCACTGTATTCTCTTTCGTTGCTCCATCCAACATCAAG |
| D181A-R | GAGAATACAGTGGTAGCACCATTTGGACCTTTGAACAGCTTCCATTTAGTACC |
| E94A-F | GCTTTGGCGAAGGTCAACAAGAAATTGTCCGACATCCAAAGAATCC |
| E94A-R | GACCTTCGCCAAAGCAACGTTGGAGTAACTCTTAACATGTCC |
| E140A-F | CCAACAACGAGTACGCAATTATGATCTGGGTTGGTTCTTACGGTGGTGC |
| E140A-R | AATTGCGTACTCGTTGTTGGAAGAAGCAGTTGGTGCCAGCCAC |
| E227A-F | GGAACTGCACCATTCTCCGGTACTAATGCACATTTCAAGACTACCG |
| E227A-R | GAGAATGGTGCAGTTCCAGCTTGCAAACTCGTAACAACCATGCCAACTG |


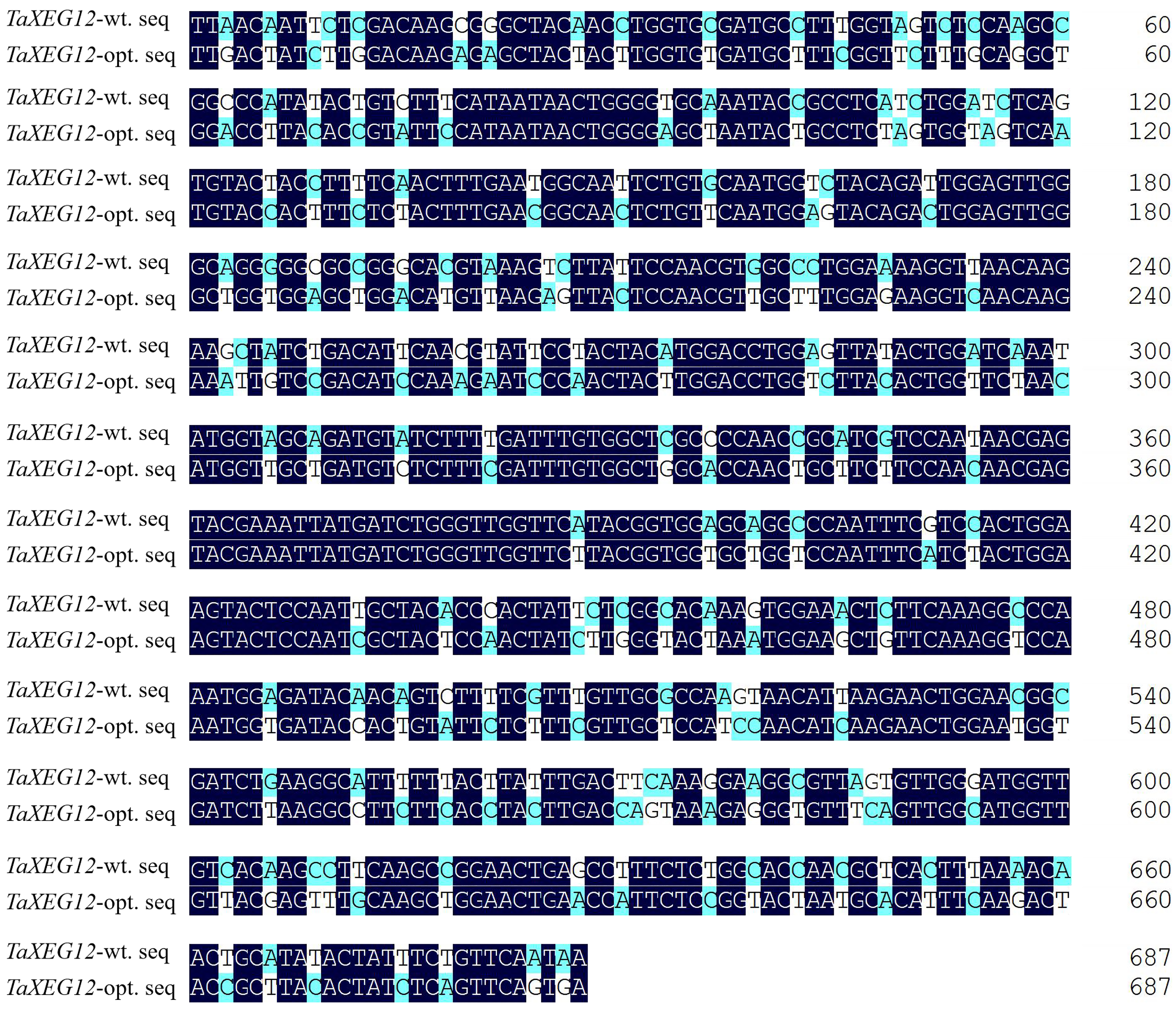


**Figure S1.** Alignment of original mature xyloglucanase gene (*TaXEG12*-wt) sequence and optimized type gene (*TaXEG12*-opt) sequence.


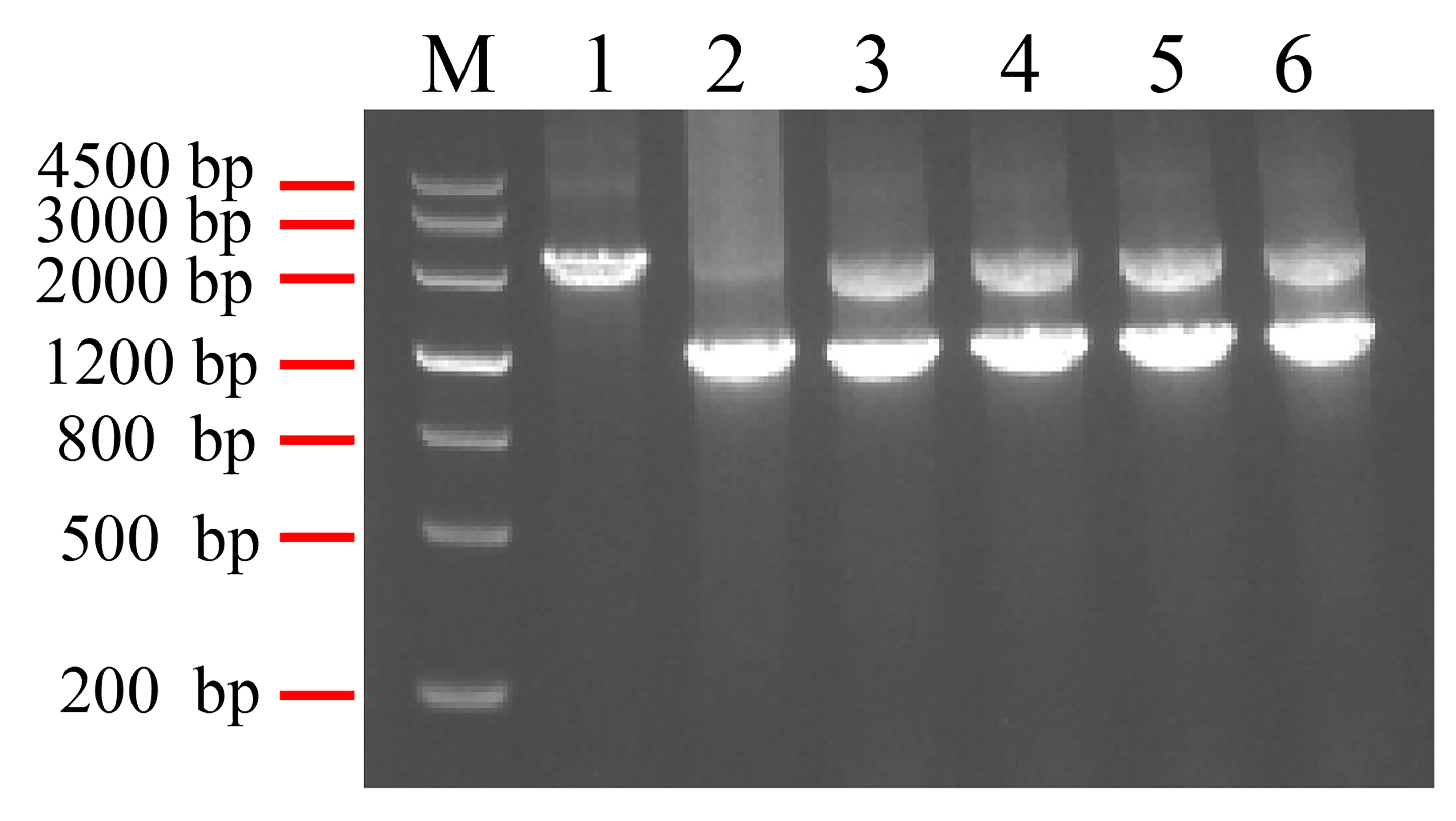


**Figure S2.** Identification of engineered strains TaXEG12-wt/TaXEG12-opt using PCR primer AOX-F/AOX-R. Lanes: M, DNA markers; 1, negative control; 2, positive control; 3-4, recombinant strain TaXEG12-wt; 5-6, recombinant strain TaXEG12-opt.
